# Airway Analysis and Morphometric Assessment of Dental Arches in Obstructive Sleep Apnea Patients

**DOI:** 10.3390/jcm14020296

**Published:** 2025-01-07

**Authors:** Domenico Ciavarella, Donatella Ferrara, Giusi Spinoso, Paolo Cattaneo, Chiara Leo, Lucio Lo Russo, Giuseppe Burlon, Carlotta Burlon, Fariba Esperouz, Michele Laurenziello, Michele Tepedino, Mauro Lorusso

**Affiliations:** 1 Department of Clinical and Experimental Medicine, University of Foggia, 71122 Foggia, Italy; domenico.ciavarella@unifg.it (D.C.); giusi_spinoso.563794@unifg.it (G.S.); chiara_leo.566312@unifg.it (C.L.); lucio.lorusso@unifg.it (L.L.R.); giuseppeburlon@gmail.com (G.B.); fariba.esperouz@unifg.it (F.E.); michele.laurenziello@unifg.it (M.L.); mauro.lorusso@unifg.it (M.L.); 2 Melbourne Dental School, Faculty of Medicine, Dentistry and Health Sciences, University of Melbourne, Melbourne, VIC 3010, Australia; paolo.cattaneo@unimelb.edu.au; 3 Department of Surgical Sciences, Postgraduate School of Orthodontics, University of Cagliari, 09124 Cagliari, Italy; carlottaburlon@gmail.com; 4 Department of Biotechnological and Applied Clinical Sciences, University of L’Aquila, 67100 L’Aquila, Italy; michele.tepedino@univaq.it

**Keywords:** obstructive sleep apnea, dental arches, morphometric analysis, CBCT

## Abstract

**Background**: Obstructive sleep apnea is a sleep-related breathing disorder associated with craniofacial morphology and dental arches. The aim of this study was to evaluate the correlation between obstructive sleep apnea and the morphometry of dental arches and upper airways. **Methods**: Forty patients were enrolled in the study, and the polysomnographic parameters evaluated were the apnea hypopnea index (AHI) and the oxygen desaturation index (ODI). Dental measurements taken from the 3D models included anterior arch widths, posterior arch widths, maxillary and mandibular arch lengths, and palatal surface area. A cone beam computed tomography (CBCT) evaluation was also performed. **Results**: In patients with moderate OSA, posterior maxillary width was significantly correlated with both minimal airway area (rho = 0.65, *p* < 0.01) and its transverse diameter (rho = 0.68, *p* < 0.01). Similarly, in patients with severe OSA, posterior maxillary width showed a significant correlation with total airway volume (rho = 1, *p* < 0.01), minimal airway area (rho = 1, *p* < 0.01), and its transverse diameter (rho = 1, *p* < 0.01). **Conclusions**: Craniofacial morphology and malocclusion can contribute to obstructive sleep apnea syndrome.

## 1. Introduction

Obstructive sleep apnea syndrome (OSAS) is a common sleep-related breathing disorder characterized by repeated episodes of apnea, which is the complete cessation of airflow for at least 10 s, or hypopnea, a significant reduction in airflow for at least 10 s, due to the collapse of the upper airway [1]. Its prevalence ranges from 9% to 38% in the general population, with a higher occurrence in males and older adults [2]. Patients with OSA exhibit a wide variety of symptoms, including nocturnal symptoms such as snoring and restless sleep, as well as daytime symptoms, the most common of which is daytime sleepiness, followed by fatigue, cognitive deficits, and behavioral disorders such as anxiety and depression [3]. OSA has a great impact on health and carries numerous consequences, especially in the long term. Studies have shown how OSA can increase the risk of cognitive impairment progressing to neurodegenerative disorders such as Alzheimer’s [4]. Among the major risks associated with OSA is cardiovascular risk. Obstructive sleep apnea appears to be an independent risk factor for the development of several diseases such as hypertension, myocardial infarction, and heart failure [5,6,7]. Studies have also shown an association between OSA and traffic accidents [8].

Obstructive sleep apnea is a very complex phenomenon associated with a multifactorial etiology. This condition is the result of the interaction between morphological alterations of the upper airways and deviations in ventilatory function during sleep [9]. In addition, OSA is also positively correlated with non-anatomical risk factors such as age, obesity, smoking, and alcohol consumption. Anatomical anomalies are the factors that contribute most to upper airway obstruction, increasing negative pressure or compromising the contraction of dilator muscles. The most recurrent anomalies concern craniofacial bone morphology, the accumulation of fat, and adeno-tonsillar hypertrophy [9,10]. The main alterations to craniofacial bone morphology include an increase in anterior facial height, a reduction in the size of the maxilla and mandible, the retropositioning of the tongue, an increase in the length and thickness of the soft palate, and the more inferior positioning of the hyoid bone [11]. Also, mandibular divergence and both mandibular and maxillary length are associated with the risk of OSA, especially with its severity [12]. Several studies have also demonstrated the relationship between OSAS and craniofacial skeletal structure using cephalometric variables, showing that craniofacial skeletal structure could influence sleep apnea severity [13,14].

Although the primary role of craniofacial and airway anomalies in the onset of OSA has been demonstrated in the literature, the correlation between these disharmonies and the severity of the syndrome has not yet been clarified. The aim of the present study is to evaluate the correlation between OSA, using the ODI, and maxillomandibular and airway anatomical characteristics.

## 2. Materials and Methods

Patients were recruited from the Orthodontics and Sleep Medicine Unit at the University Dental Clinic. The sample consisted of 40 male patients with a mean age of 53.5 years, all diagnosed by Home Sleep Apnea Testing (HSAT) (Embletta system X-100, Flaga, Reykjavik, Iceland), which showed that obstructions were greater in a supine position than in a non-supine position with a ratio of 2 to 1. Following diagnosis and a drug-induced sleep endoscopy set using the Marsh model with Diprivan (Propofol) at 2% (DISE), each patient underwent therapy with a mandibular advancement device (MAD). DISE is a valuable procedure for patients with sleep-disordered breathing, used to assess the upper airways in individuals with obstructive sleep apnea (OSA). It is typically employed to guide treatment decisions for OSA [15,16,17]. DISE showed that obstructions occurred anteroposteriorly in retropalatal and -oral sites. The enrolled patients presented a Mallampati score of a first or second stage: the authors used the Vicini upper airway obstruction classification to stage the site of obstruction and its severity. All patients were evaluated by an otolaryngologist to exclude patients with septal deviation, turbinate hypertrophy, and adenoids. The authors used both the NOHL and VOTE classifications. 

The inclusion criteria were the following: an age greater than 18 years old, a body mass index (BMI) lower than 33.9 kg/m^2^, and a diagnosis of moderate-to-severe OSA (AHI > 15). Patients with a smoking habit, periodontitis or tooth loss, any comorbidities such as cardiovascular or pulmonary diseases, previous cervical trauma or neurological disorders, 5 < AHI < 15, and a tongue posture and morphology comparable to stage IV of Friedman were excluded from this study.

Pre-treatment polysomnography, 3D models of the dental arches, and pre-treatment CBCT were examined for each patient.

Participants underwent split-night polysomnography (SN-PSG) with a minimum of 4 h of continuous recording in a sleep laboratory, using a Type 2 portable device (Embletta system X-100, Flaga, Reykjavik, Iceland).

### 2.1. Polysomnographic Parameters

The patient’s pre-treatment polysomnography parameters were the body mass index (BMI), the apnea–hypopnea index (AHI), and the oxyhemoglobin desaturation index (ODI). According to the AHI, the sample was divided into two groups: moderate OSA (15 < AHI < 30) and severe OSA (AHI > 30). The ODI indicates the number of 4% oxygen desaturations.

### 2.2. Three-Dimensional Dental Models’ Evaluation

**Figure 1:**
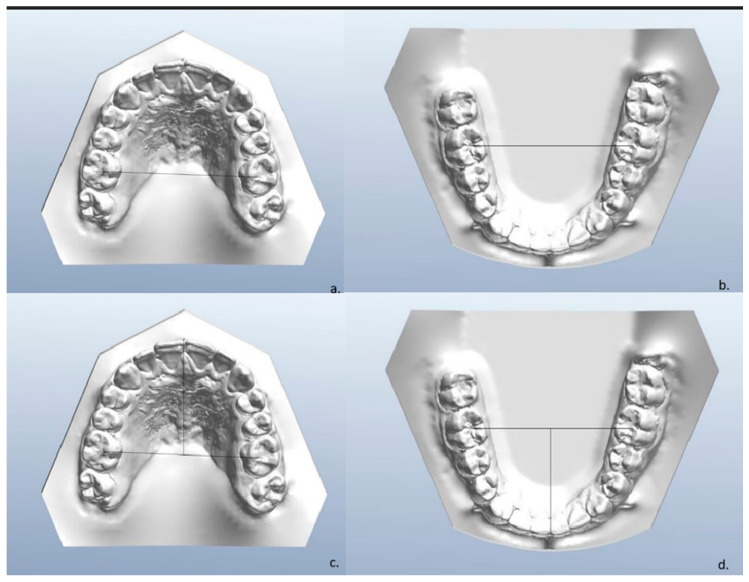
(**a**) Posterior width of upper arch. (**b**) Posterior width of lower arch. (**c**) Upper arch length. (**d**) Lower arch length.

**Figure 2:**
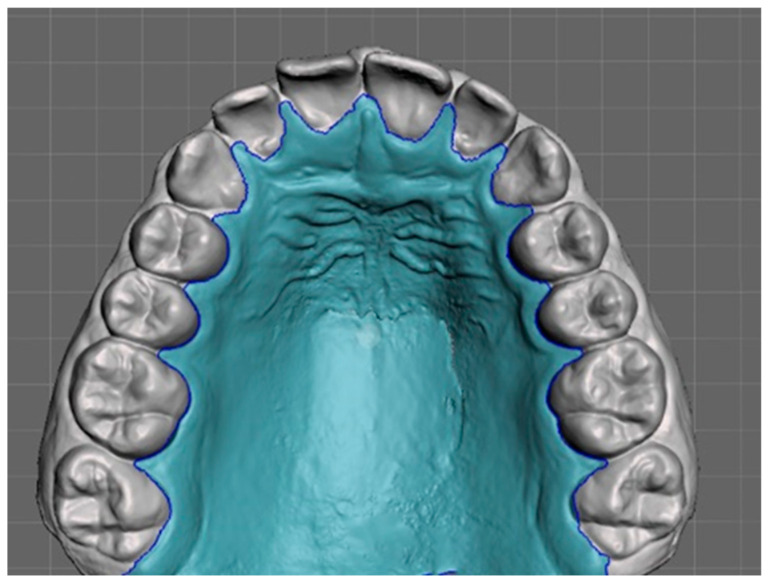
Palatal surface area.

Dental impressions were scanned with the intraoral scanner TRIOS 3 (3Shape, Copenhagen, Denmark). Three-dimensional models were analyzed using Ortho Viewer (2017) and Autodesk Mesh mixer software 2.5. The measurements analyzed were the following:

- Posterior maxilla widths, defined as the distance between the central groove of the upper and lower first molars (Figure 1a,b);
- Maxillary and mandibular arch lengths, defined as the distance between the most labial point of the central incisors and a line passing through the central fossae of the first molars (Figure 1c,d);
- Palatal surface area, defined as the palatal area isolated by the rest of the model and expressed in mm^2^ (Figure 2)

### 2.3. CBCT Evaluation

**Figure 3:**
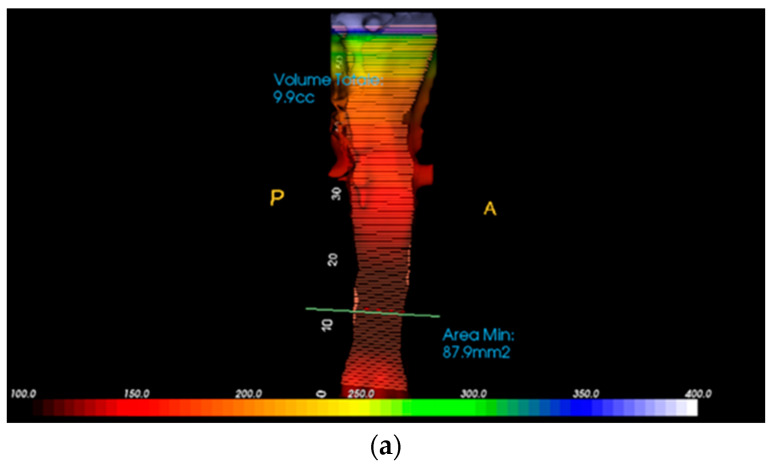

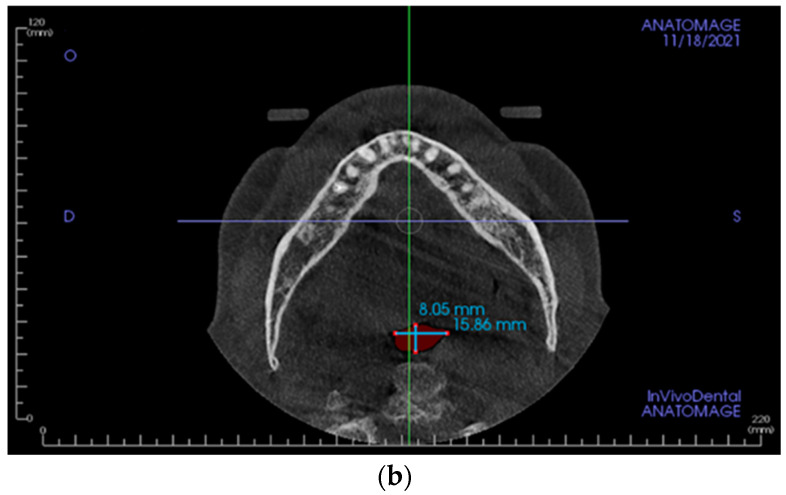
(**a**) Total airway volume and minimum airway area. (**b**) Minimum cross-section of the airway and calculation of its sagittal and transversal diameters. (Posterior (P), Anterior (A), Right (D), Left S).

Each patient in the study underwent a pre-treatment cone beam computed tomography (CBCT) scan, performed before 2020. DICOM files were imported into InVivo Dental software 6.0 to analyze the airways, and to obtain the total volume in cm^3^ and the minimum area in mm^2^. This piece of software creates a visual representation of airway volumes using a color code and indicates the cross-section of the airways in red. Furthermore, the sagittal and transverse diameters were calculated at the minimum cross-section of the air lumen (Figure 3a,b).

### 2.4. Statistical Analysis

This study was conducted in accordance with the Strengthening the Reporting of Observational Studies in Epidemiology (STROBE) guidelines [18]. All procedures outlined in the research protocol complied with the ethical principles of the Declaration of Helsinki and received approval from the Ethics Committee of the University of Foggia (Approval No. 43/CE/2019). Patient records were retrospectively retrieved and anonymized for analysis, and all participants provided written informed consent.

The random error of each measurement was calculated using Dahlberg’s formula (S = ∑ d2/2N), where d is the difference between the first and second measurements, and N is the number of radiographs evaluated [19,20]. The random error of measurements ranged from 0.01 to 0.05 for linear measurements, from 0.2 to 0.4 for area measurements, and from 0.01 to 0.03 for volumetric measurements.

A power analysis (G*Power 3.1.9.2, Franz Faul, Universitat Kiel, Kiel, Germany) revealed that to detect a large effect size of 0.5 [21] with a Spearman test, an α error probability of 0.05, and a power (1 − β error probability) of 0.95, 32 subjects would be needed.

Data were analyzed using GraphPad Prism software 6.0 (GraphPad Prism Software, San Diego, CA, USA). The Shapiro–Wilk normality test was conducted to evaluate data distribution. As the variables failed the normality test, a Spearman rho test was used to analyze the correlation between the respiratory indexes and dental variables. Descriptive statistics are reported in Table 1 and Table 2.

## 3. Results

**Figure 4:**
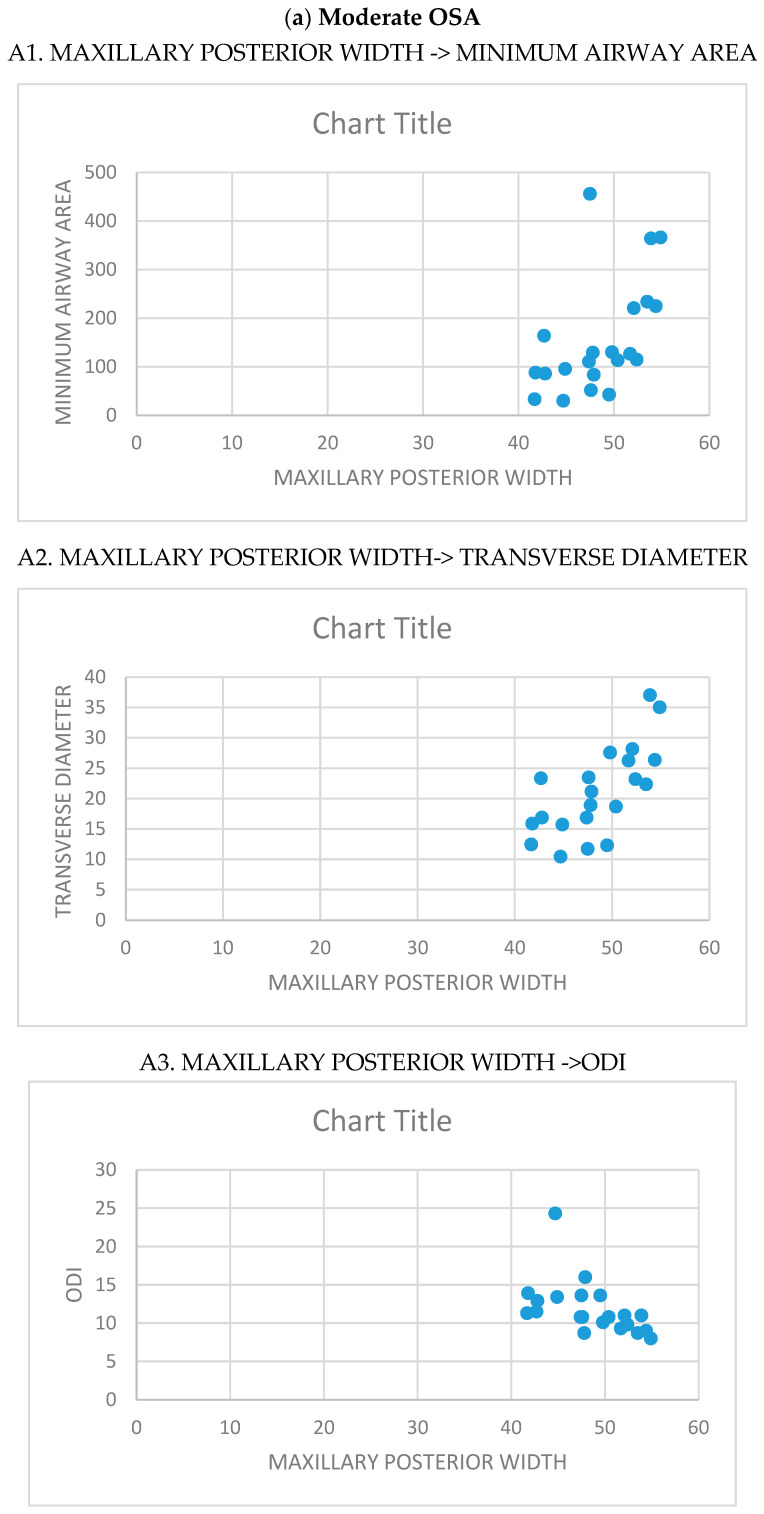

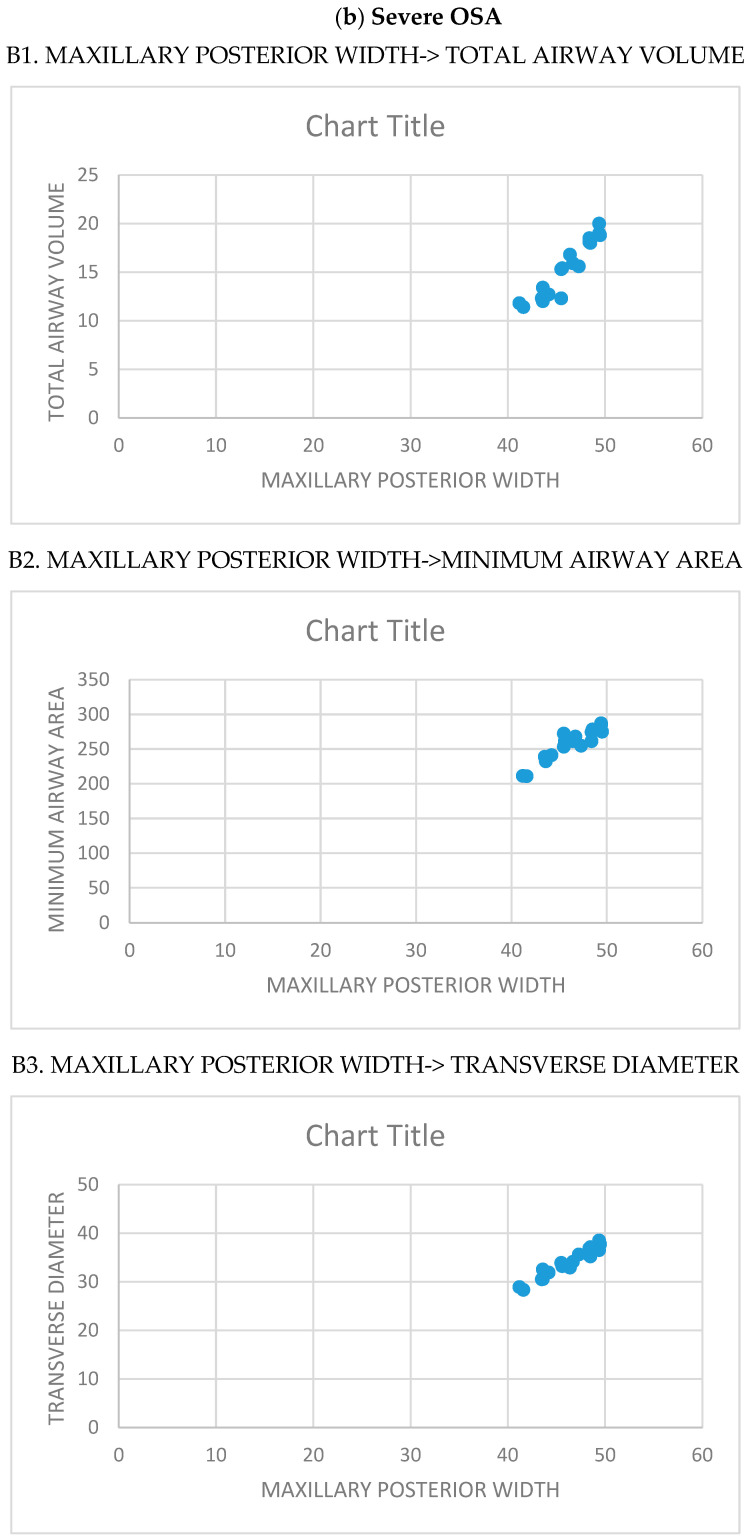

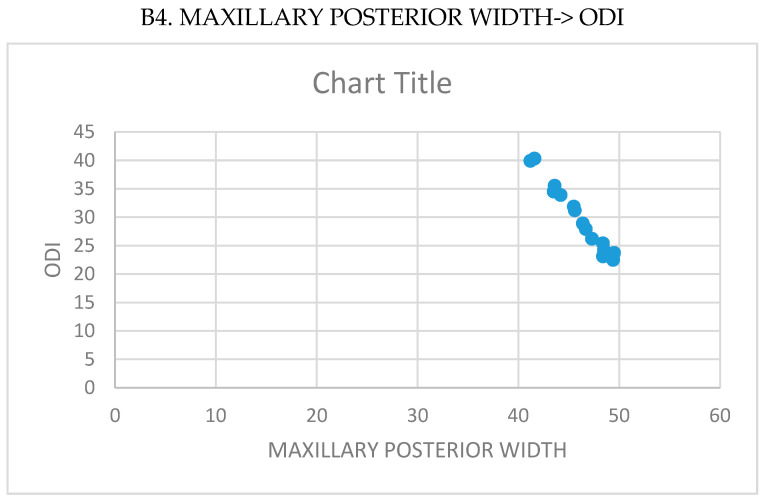
Graphic demonstration of the correlations.

The results of the Spearman test are reported in Table 3 and Table 4. In the moderate OSA group, statistically significant correlations emerged between the posterior width of the maxilla (measured as the upper inter-molar distance) and both the minimum airway area and the transverse diameter of the minimum airway area. Moreover, the posterior width of the maxilla shows a statistically significant inverse correlation with the ODI. In the severe OSA group, the posterior width of the maxilla shows statistically significant correlations with the total airway volume, the minimum air area, and the transverse diameter of the minimum air area, and an inverse correlation with the ODI. Furthermore, the total air volume, the minimum air area, and the transversal diameter of the minimum air area show a statistically significant inverse correlation with the ODI. Therefore, in the severe OSA group, the posterior width of the maxilla shows both a direct and transversal correlation with the ODI. In the severe OSA group, a statistically significant inverse correlation was observed between the mandibular arch length and the ODI. Correlations are shown in Figure 4.

## 4. Discussion

The role of cranio-maxillary and airway anomalies in the pathogenesis and severity of obstructive sleep apnea syndrome has been widely debated in the literature. Indeed, many treatment plans focus on correcting and improving these anatomical disharmonies [22,23,24]. The abnormalities most often associated with apnea include mandibular deficiency, an inferiorly positioned hyoid bone relative to the mandibular plane, a narrow posterior airspace, and the elongation of the soft palate [25]. However, there are no studies that have simultaneously correlated the severity of OSA with the anatomical characteristics of the upper airways and maxillary arches.

Some research in the literature has explored the anatomy of the airways of patients with OSA using cone beam computed tomography (CBCT), and most authors agree on the fact that the airways appear narrower and smaller in patients with obstructive sleep apnea, especially in a severe form. In a study conducted by Wen et al. [26], it was observed that patients with severe OSA had upper airways that were narrower with a reduced volume compared to patients with a milder form. Ogawa et al. also found a smaller air volume in patients with OSA, although it was not statistically significant. Furthermore, the group with OSA tended to have concave or elliptical airways [27].

Regarding the correlations between dental arch shape and OSAS, previous studies are limited and focus exclusively on the upper jaw. Kecik compared palatal and airway morphology between patients with OSA and a control group and found that the group with OSA had a narrower jaw, as well as reduced inter-canine and inter-molar distances [28]. In line with the results of Kecik, Seto et al. also found a significant reduction in inter-canine, inter-premolar, and inter-molar distances of the maxilla in patients with OSA [25]. Johal et al. examined the morphology of the maxilla using a lateral cephalogram and study models of the arches, finding an increased palatal height in patients with OSA [29].

Only a few studies, such as those by Irlandese et al. [30] and Ciavarella et al. [31], also examined the lower arch. Irlandese et al. found different dental arch shapes between patients with OSA and controls. Additionally, patients with OSA showed reduced inter-canine, inter-premolar, and inter-molar widths for both the maxilla and the mandible [30]. The results of the present study highlighted a worsening of the AHI and the ODI, and therefore an increase in the severity of OSA, in association with a significant reduction in maxillary arch length and mandibular anterior width. These findings suggest that a contracted dental arch is a risk factor for OSA, as it lowers the position of the tongue, thereby promoting the narrowing of the retrolingual space.

Moreover, a significant inverse correlation was found between the ODI and palatal area and height. Therefore, the inverse correlation between the ODI and palatal height may be due to the reduced palatal area, which causes the tongue to move backwards and downwards, leading to a narrowing of the upper airways during sleep. In line with the results of the present study, Irlandese et al. [30] found a significant reduction in the lower inter-canine width in patients with OSA. Johal et al. [29] found no differences in the anterior and posterior interdental widths of the maxilla, in contrast to the results of Kecik and Irlandese et al. [28,30], who reported a significant reduction in maxillary inter-molar and inter-canine distances in patients with OSA. Furthermore, according to Kecik [28], a 3D analysis of the palatal surface revealed a significant reduction in palatal volume in patients with OSA, demonstrating the association between palatal morphology and the pharyngeal airways. In the current study, in the moderate OSA group, a statistically significant correlation was highlighted between the posterior width of the upper arch and the transverse diameter of the minimum air area and the minimum air area itself. Therefore, when the posterior width of the maxilla decreases, both the minimum area of the upper airways and the transverse diameter of this area are reduced, predisposing the individual to the condition. Furthermore, maxillary posterior width shows a significant inverse correlation with the ODI, meaning that a reduced posterior width of the maxilla is associated with a higher ODI value. Therefore, the maxilla posterior width presents a double correlation with the ODI, both directly and through the airway parameters, reinforcing what was found in the moderate OSA group. These results suggest that maxilla posterior width could be considered a relevant factor in the severity of obstructive sleep apnea syndrome. A statistically significant correlation between the posterior width of the maxillary arch and both the minimum airway area and the transverse diameter of the minimum airway area was also observed in the group with severe OSA. Furthermore, a significant correlation with total airway volume was also found. Total airway volume, the minimum airway area, and the transverse diameter of this area show a negative correlation with the ODI. Indeed, as these parameters decrease, the severity of the condition increases. Another interesting result is the inverse correlation between lower arch length and the ODI, suggesting that a smaller mandibular size may be associated with a greater severity of obstructive sleep apnea syndrome. Other studies did not find any correlation between maxillary variables and OSA severity, but some measurements, such as maxillary transverse diameter, could predict the obstruction of the upper airways during DISE [32,33].

In fact, DISE is an excellent tool for airway assessment during sleep in patients with OSA. Studies have shown that the anatomy of soft tissues and their volumetry, especially of the tongue, assessed by awake CT can also influence upper airway collapse during sleep, and thus, during a DISE examination, this correlation can be demonstrated [34].

### Limitations of the Study

CBCT images were acquired with patients in an upright position and awake. Given the dynamic nature of the upper airway, variations due to respiratory artifacts, swallowing, and head position may have affected the measurements. Another limitation is the study’s focus on male patients only. Finally, the small sample size is also a limitation.

## 5. Conclusions

Craniofacial anatomy plays a crucial role in the development of obstructive sleep apnea. The alterations not only affect the airway but also affect dental malocclusions.

In the present study, patients with moderate OSA exhibited a significant correlation between posterior maxillary width and both the minimal airway area and the transverse diameter of the minimal airway area. Additionally, posterior maxillary width showed a statistically significant inverse correlation with the ODI. In patients with severe OSA, posterior maxillary width showed a significant correlation with total airway volume, the minimal airway area, and the transverse diameter of the minimal airway area, as well as an inverse correlation with the ODI. Furthermore, total airway volume, the minimal airway area, and the cross-sectional diameter of the minimal airway area showed a statistically significant inverse correlation with the ODI.

A narrow maxillary arch could cause airway restriction and consequently reduce airflow in patients with obstructive sleep apnea. Future studies are essential to understand the highlighted morphometric associations.

## Figures and Tables

**Table 1:** Descriptive statistics of the variables analyzed in the group with moderate OSA.

Cohort (n = 21)	Mean	Median	95% Confidence Interval for the Mean		Standard Deviation	Passed Normality Test?
			Inferior Limit	Upper Limit		
Total airway volume	9	7.2	6.95	11.04	3.83	NO
Minimum airway area	133.98	118.67	76.44	191.51	107.97	NO
Sagittal diameter	6.34	5.78	5.23	7.45	2.08	NO
Transverse diameter	21.41	15.26	16.80	26.02	4.65	NO
Palatal surface	2335.62	2147.84	2207.31	2463.93	240.79	NO
Maxillary length	33.21	30.71	31.23	35.19	3.71	NO
Mandibular length	31.60	26.19	27.74	35.45	5.22	NO
Maxillary posterior width	48.32	36.32	46.16	50.48	4.06	NO
Mandibular posterior width	44.61	39.17	40.56	48.87	3.61	NO
ODI	17.78	15.68	13.38	22.19	2.27	NO
AHI	22.78	21.47	19.37	26.18	6.39	NO

**Table 2:** Descriptive statistics of the variables analyzed in the group with severe OSA.

Cohort (n = 19)	Mean	Median	95% Confidence Interval for the Mean		Standard Deviation	Passed Normality Test?
			Inferior Limit	Upper Limit		
Total airway volume	13.42	14.23	10.12	16.72	2.07	NO
Minimum airway area	188.25	189.17	32.62	343.87	97.8	NO
Sagittal diameter	8.1	7.53	5.16	11.04	1.84	NO
Transverse diameter	23.33	28.91	10.37	36.39	6.14	NO
Palatal surface	2310.99	2315.86	2248.68	2373.29	39.15	NO
Maxillary length	32.22	31.29	27.09	37.35	3.22	NO
Mandibular length	27.85	27.71	26.94	28.75	1.56	NO
Maxillary posterior width	46.97	45.18	42.35	51.59	2.9	NO
Mandibular posterior width	40.72	39.69	36.96	44.48	2.36	NO
ODI	34	26.87	4.36	49.63	3.22	NO
AHI	45.37	44.16	26.69	64.05	11.74	NO

**Table 3:** Spearman rho correlation test in the group with moderate OSA.

	Total Airway Volume	Minimum Airway Area	Sagittal Diameter	Transverse Diameter	Palatal Surface	Maxillary Length	Mandibular Length	Maxillary Posterior Width	Mandibular Posterior Width	ODI
Total airway volume	1	0.89 **	0.82 **	0.83 **	0.35	−0.29	0.26	0.46	0.28	0.27
Minimum airway area	0.89 **	1	0.62 *	0.90 **	0.24	−0.59 *	0.18	0.65 **	0.40	0.34
Sagittal diameter	0.82 **	0.62 *	1	0.28	0.22	−0.05	0.34	0.21	−0.06	0.38
Transverse diameter	0.83 **	0.90 **	0.28	1	0.47	−0.54 *	0.14	0.68 **	0.50 *	0.22
Palatal surface	0.35	0	0.22	0.47	1	0.30	0.15	0.59 *	0.70 **	0.54 *
Maxillary length	−0.29	−0.59 *	−0.05	−0.54 *	0.30	1	−0.05	−0.32	0.02	0.05
Mandibular length	0.26	0.18	0.34	0.14	0.15	−0.05	1	−0.24	−0.38	0.24
Maxillary posterior width	0.46	0.65 **	0.21	0.68 **	0.59 *	−0.32	−0.24	1	0.85 **	−0.63 **
Mandibular posterior width	0.28	0.40	−0.06	0.50 *	0.70 **	0.02	−0.38	0.85 **	1	0.48
ODI	0.27	0.34	0.38	0.22	0.54 *	0.05	0.24	−0.63 **	0.48	1

* *p* < 0.05, ** *p* < 0.01.

**Table 4:** Spearman rho correlation test in the group with severe OSA.

	Total Airway Volume	Minimum Airway Area	Sagittal Diameter	Transverse Diameter	Palatal Surface	Maxillary Length	Mandibular Length	Maxillary Posterior Width	Mandibular Posterior Width	ODI
Total airway volume	1	1 **	−0.77	1 **	0.77	0.33	0.33	1 **	−0.77	−1 **
Minimum airway area	1 **	1	0.77	1 **	0.77	0.33	0.33	1 **	0.77	−1 **
Sagittal diameter	−0.77	−0.77	1	−0.77	−1 **	0.33	0.33	0.77	1 **	0.77
Transverse diameter	1 **	1 **	−0.77	1	0.77	0.33	0.33	1 **	0.77	0.77
Palatal surface	0.77	0.77	−1 **	0.77	1	−0.33	−0.33	−0.77	−1 **	−1 **
Maxillary length	0.33	0.33	0.33	0.33	−0.33	1	1 **	−0.33	0.33	−0.33
Mandibular length	0.33	0.33	0.33	0.33	−0.33	1 **	1	−0.33	0.33	−0.95 *
Maxillary posterior width	1 **	1 **	0.77	1 **	−0.77	−0.33	−0.33	1.00	0.77	−1 **
Mandibular posterior width	−0.77	−0.77	1 **	−0.77	−1 **	0.33	0.33	0.77	1	0.77
ODI	−1 **	−1 **	0.77	−1 **	−0.77	−0.33	−0.95 *	−1 **	0.77	1

* *p* < 0.05, ** *p* < 0.01.

## Data Availability

The original contributions presented in the study are included in the article, further inquiries can be directed to the corresponding authors.
